# Pilot Study of Voxel-Based Morphometric MRI Post-processing in Patients With Non-lesional Operculoinsular Epilepsy

**DOI:** 10.3389/fneur.2020.00177

**Published:** 2020-03-19

**Authors:** Wei Wang, Qilin Zhou, Xiating Zhang, Liping Li, Cuiping Xu, Yueshan Piao, Siqi Wu, Yajie Wang, Wei Du, Zhilian Zhao, Yicong Lin, Yuping Wang

**Affiliations:** ^1^Department of Neurology, Xuanwu Hospital, Capital Medical University, Beijing, China; ^2^Department of Neurosurgery, Xuanwu Hospital Capital Medical University, Beijing, China; ^3^Department of Pathology, Xuanwu Hospital Capital Medical University, Beijing, China; ^4^Department of Radiology, Xuanwu Hospital Capital Medical University, Beijing, China; ^5^Beijing Key Laboratory of Neuromodulation, Beijing, China; ^6^Center of Epilepsy, Beijing Institute for Brain Disorders, Capital Medical University, Beijing, China

**Keywords:** epilepsy, operculoinsular, MRI-negative, voxel-based morphometry, magnetoencephalography

## Abstract

**Objective:** The aim of this study was to use voxel-based MRI post-processing in detection of subtle FCD in drug-resistant operculoinsular epilepsy patients with negative presurgical MRI, and by combining magnetoencephalography (MEG) to improve the localization of epileptogenic zone.

**Methods:** Operculoinsular epilepsy patients with a negative presurgical MRI were included in this study. MRI post-processing was performed using a Morphometric Analysis Program (MAP) on T1-weighted volumetric MRI. Clinical information including semiology, MEG, scalp electroencephalogram (EEG), intracranial EEG and surgical strategy was retrospectively reviewed. The pertinence of MAP-positive areas was confirmed by surgical outcome and pathology.

**Results:** A total of 20 patients were diagnosed with operculoinsular epilepsy had non-lesional MRI during 2010–2018, of which 11 patients with resective surgeries were included. MEG showed clusters of single equivalent current dipole (SECD) in inferior frontal regions in five patients and temporal-insular/ frontal-temporal-insular/parietal-insular regions in five patients. Four out of 11 patients had positive MAP results. The MAP positive rate was 36.4%. The positive regions were in insular in one patient and operculoinsular regions in three patients. Three of the four patients who were MAP-positive got seizure-free after successfully resect the MAP-positive and MEG-positive regions (the pathology results were FCD IIb in two patients and FCD IIa in one patient).

**Conclusions:** MAP is a useful tool in detection the epileptogenic lesions in patients with MRI-negative operculoinsular epilepsy. Notably, in order to make a right surgical regime decision, MAP results should always be interpreted in the context of the patient's anatomo-electroclinical presentation.

## Introduction

In patients with drug-resistant epilepsy, complete surgical resection of the epileptogenic zone can be an effective treatment. Detection and delineation of the epileptogenic lesion are essential to the success of epilepsy surgery. While magnetic resonance imaging (MRI) may identify a cause in many patients (e g., tumors, traumatic scars, and vascular malformations), approximately one-third have no clear epileptogenic lesions (i.e., are MRI-negative or non-lesional) (1). Absence of a structural lesion on MRI represents a major challenge for surgical management. MRI-negative patients typically require expensive and invasive intracranial electroencephalogram (ICEEG), and tend to have poorer seizure outcomes (2, 3).

Although operculoinsular epilepsy is relatively rare, it accounts for a non-negligible proportion of drug-resistant epilepsy surgical candidates and sometimes it can be difficult to be recognized (4–6). Because of the complex physiology and the rich connections to surrounding and remote structures, the clinical manifestations of operculoinsular seizures can be diverse (7). It is reported that operculoinsular epilepsy can have semiology similar to temporal lobe seizures, frontal lobe seizures or parietal lobe seizures (8–13). The apparent heterogeneous and sometimes non-specific clinical manifestations increase the difficulties of the diagnosis of operculoinsular seizures, especially in non-lesional patients. Lack of recognition of operculoinsular seizures may be responsible for some epilepsy surgery failures (6).

Recent advances in neuroimaging and image processing have consistently demonstrated that a negative MRI by unaided visual analysis may not be truly negative (14). Subtle lesions can escape routine visual inspection, especially when non-invasive evaluation data, such as scalp eletroencephalogram (EEG) and semiology, do not point to a specific area of interest. A number of studies showed that a voxel-based MRI morphometric analysis program (MAP) is a useful tool for identifying subtle lesions (15–22). Because of the high incidence, previous MAP studies mainly focus on patients with frontal lobe epilepsy and temporal lobe epilepsy (18). The report of operculoinsular epilepsy is very limited. Here, we summarize the clinical data of patients with drug-resistant insular epilepsy with a negative MRI by visual analysis in our epilepsy center and use voxel-based MRI post-processing to improve detection of subtle focal cortical dysplasia (FCD) in these patients.

## Patients and Methods

### Patients

We reviewed the clinical data of patients who were diagnosed as operculoinsular epilepsy from our surgical database from December 2010 to August 2018 at Xuanwu Hospital, Capital Medical University, which is a large tertiary epilepsy center in China. This study was approved by the Research Ethics Committee of Xuanwu Hospital, Capital Medical University. Written informed consent was obtained from each subject (for pediatric patients, legal guardians of the patients signed the informed consent). Patients were included in the study if they: (1) had epilepsy resective surgery; (2) had a preoperative 3T MRI with T1-weighted (T1w) magnetization prepared rapid acquisition with gradient echo (MPRAGE) sequence; (3) had negative MRI by radiology report; (4) were diagnosed as insular or operculoinsular epilepsy; (5) had >12 months post-surgical follow-up. Patients were excluded if: (1) the MRI was of poor quality; (2) preoperative MRI showed a lesion in operculoinsular regions; (3) the follow-up was <12 months.

The strategies for ICEEG implantation and surgical resection of all patients were made during a multidisciplinary patient management conference, based on a combination of all the non-invasive data, including semiology, scalp EEG, positron emission tomography (PET), and magnetoencephalography (MEG). MEG data was recorded from a 306-channel whole-head MEG system (Elekta, Helsinki, Finland), and individual spike source localization was performed on data segments containing visually identified epileptiform discharges using single equivalent current dipole model (SECD) (23).

### Surgical Outcome and Pathology

The results of surgical outcomes at 12 months were assessed using modified Engel's classification (24). Patients were considered completely seizure-free (Engel's class Ia) if they didn't have any seizure or aura at the 12 months follow-up after surgery. Otherwise, the patients were considered not seizure-free (Engel's class Ib—IV). The classification of FCD was in accordance with the International League Against Epilepsy classification (25).

### MRI Post-processing

MAP was carried out using SPM12 (Wellcome Department of Cognitive Neurology, London, UK) in MATLAB 2015a (MathWorks, Natick, MA) following previously established methods (15, 18, 20, 26) and same as our previous procedures (27). The clinical MRI protocol in our center for epilepsy patients includes 3D T1w MPRAGE sequence, T2-weighted (T2w) turbo spin-echo (TSE) sequences and T2w fluid-attenuation inversion recovery (FLAIR) acquisition. MAP was performed on T1w MPRAGE images. For each patient, the computed output junction images, which highlight brain structures deviating from the average normal brain, and may therefore indicate the presence of subtle lesion. The junction image is sensitive to blurring of the gray-white matter junction. MAP was performed using the z-score threshold of 4 to identify candidate MAP-positive regions on the junction file, thickness file and extension file. High z-score areas due to artifacts and non-specific white matter inhomogeneity were excluded. All the MAP results were reviewed by two independent reviewers (Wang W and Lin Y).

## Results

### Cohort Summary

A total of 20 patients were diagnosed with operculoinsular epilepsy had non-lesional MRI during 2010–2018 in our epilepsy center, of which 12 patients had resective surgeries. One patient was excluded because of poor MRI quality. Eleven patients were included in this study. All patients were reviewed during the multidisciplinary patient management conference and considered MRI-negative before surgery.

The clinical characteristics and surgical outcomes of these patients were summarized in Table 1. Seizure semiology, scalp EEG, invasive EEG, MEG results were also shown in Table 1. Six out of 11 patients were female (54.5%) and the average age was 18.5 years (median: 15, range: 13–32).

**Table 1:** Clinical characteristics and surgical outcomes of the MRI-negative patients.

**Patient No**.	**Age**	**Sex**	**Epilepsy Duration/years**	**Seizure semiology**	**Scalp EEG**		**Invasive EEG**		**MEG**	**MAP**	**Surgery**	**Surgical outcome (Engel's classification)**	**Pathology**
					**Interictal ED**	**Seizure onset**	**Place of ICE**	**Seizure onset**					
1	18	Female	7	Non-specific aura → oral automotor seizure → L hemi tonic seizure	R Sphenoidal	R-T, F, C(maximal F4)	R-F, I, H	R-I, H	R- inferior F	Negative	R-mesial T, anterior I, F opercular	Ia	FCD Ic
2	15	Male	8	Sensory aura (R paresthesiae) → R hemi tonic seizure	L-F; R-F, Vertex	Probably lateralized L	L-F, I, P, H	L-I, H	L-C (sparsely)	Negative	L-I, I opercular, mesial T	Ia	FCD Ic
3	22	Female	11	Non-specific aura → L hemi tonic clonic seizure → hypermotor seizure	Normal	Non-localizable	R- F, I, P, H	R-I	R- inferior F	R-I	R-I opercular, I	Ia	FCD IIb
4	15	Female	12	Sensory aura (thoracic constriction) → oral automatism → L hemi tonic clonic	R-F, C; B-T, P, O	R-F, C	R-I, F, C	R-I	R- inferior F	Negative	R-F opercular, I	IIIa	FCD IIa
5	13	Female	6	Abdominal aura → L eye deviation → L versive head turn → L hemi tonic	B-F	R-F, T	R-I, H, T	R-I	R- inferior F	R-I opercular	R-I opercular, I	Ia	FCD IIa
6	15	Female	13	1, Asymmetric tonic (L arm extension, R arm flexion, bilateral leg tonic); 2, staring	generalized, maximal R	Non-localizable	R-F, P, T, I	R-F, P, C, I	R- inferior F	R-F opercular	R-F opercular	IIIa	FCD IIa
7	24	Male	24	Sensory aura → R hemi clonic	L-F, T, C	L-F, C, P	L-F, T, C, I	L-I, P opercular, T opercular,	L-T	L-F, T opercular	L-opercular (P opercular and T opercular)	Ia	FCD IIb
8	20	Male	11	1, Fear sensation → bilateral tonic with retained awareness; 2, sensory aura → L eye deviation or vocalization	R-F, T, C	R-F, T, C	1^st^ surgery: R-F, I, SMA, CG; 2^nd^ surgery: R-F, T	1^st^ surgery: R-F, T; 2^nd^ surgery: R-F, T	R-F, T	Negative	1^st^ surgery: R-F, F opercular, anterior CG; 2^nd^ surgery: R- F, T (including H)	Ia	1^st^ surgery: FCD I; 2^nd^ surgery: FCD IIa
9	14	Male	2	Abdominal aura → R face clonic → GTCS or L hand automatism	L-T	L-T	L-F, anterior and middle I, T	L-F	L-I, T	Negative	L-IFG, anterior I	Ia	FCD IIa
10	15	Male	6	Viscerosensory aura → bilateral complex motor → vocalization	B-F, T (maximal R)	R-T	R-F, T	R-F	R-C, I	Negative	R-F, anterior I	Ia	FCD Ib
11	32	Female	8	1, Abdominal aura → R hand automatism → staring; 2, GTCS	B-T (maximal R)	B-F	R-F, T, I	R-F, T	R-F, I, STG	Negative	R-F, anterior T, I	Ia	FCD I

*ED, epileptiform discharges; EEG, electroencephalogram; SF, seizure free; MEG, magnetoencephalography; FCD, focal cortical dysplasia; R, right; L, left; B, bilateral; ATL, anterior temporal lobectomy; F, Frontal lobe; T, Temporal lobe; C, central; P, parietal lobe; O, occipital lobe; I, insula; H, hippocampal; ICE, intracranial electrodes; CG, cingulate gyrus; IFG, inferior frontal gyrus; STG, superior temporal gyrus; GTCS, generalized tonic clonic seizure; SMA, supplementary motor area*.

All patients were right-handed. The mean duration of epilepsy was 9.8 years (median: 8, range: 2–24). All patients underwent a 3T scan. The mean follow-up was 51.5 months (median: 34, range: 12–104).

### Seizure Semiology

Ten out of 11 (90.9%) patients had aura, of which two had non-specific aura, one had somatosensory aura, one had fear sensation, and six had viscerosensory aura such as abdominal aura and thoracic constriction. The seizure may evolve to tonic seizure, clonic seizure, automotor seizure, or complex motor seizure, as shown in Table 1.

### MEG and Invasive EEG

**Figure 1:**
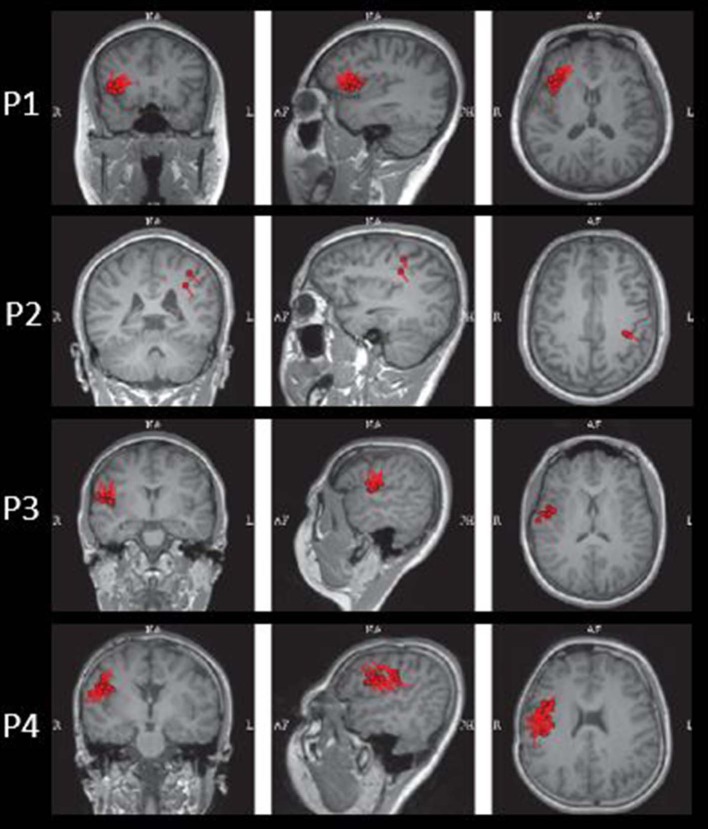
The MEG results of Patient 1–4 in coronal, sagittal, and axial images.

**Figure 2:**
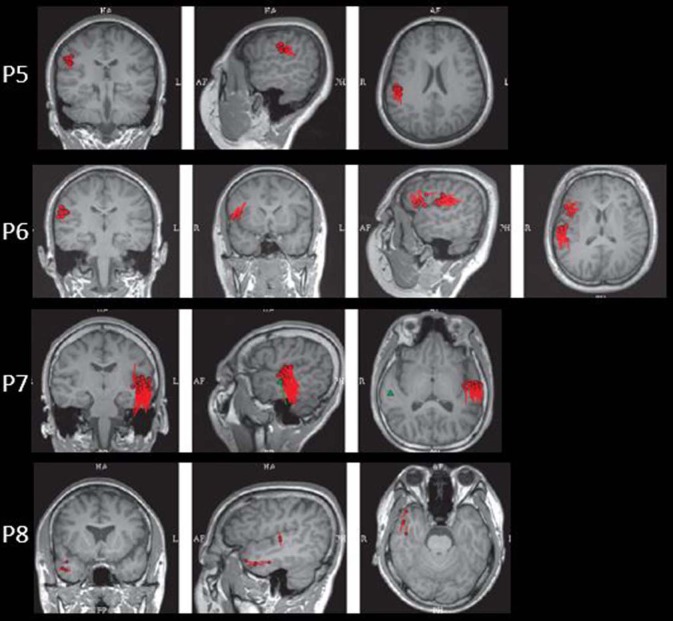
The MEG results of Patient 5–8 in coronal, sagittal, and axial images.

**Figure 3:**
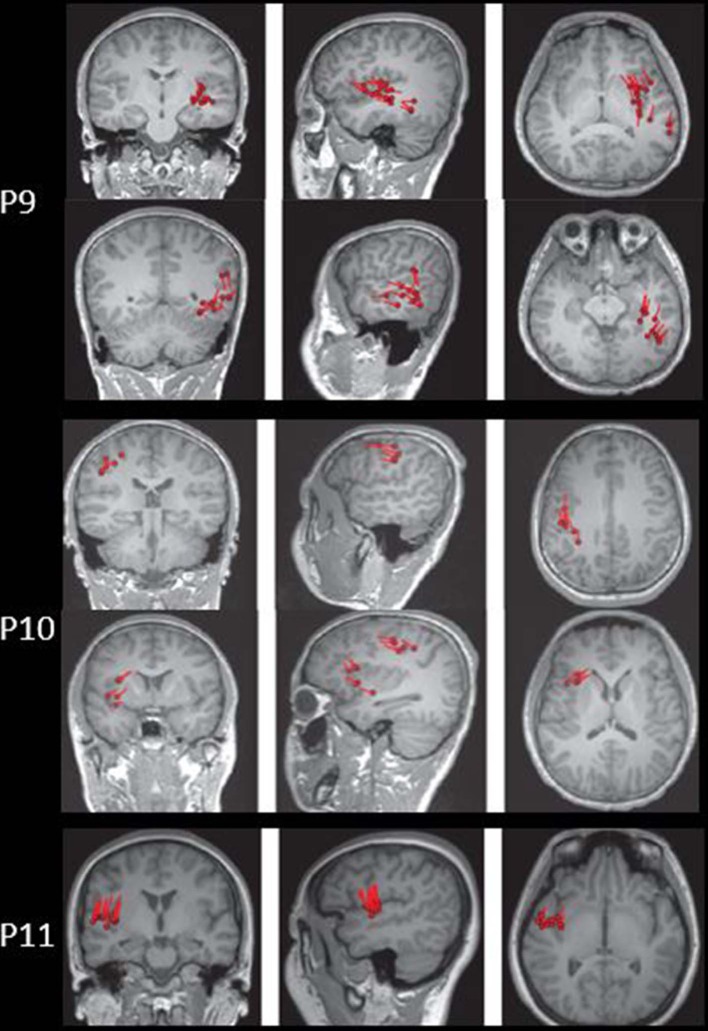
The MEG results of Patient 9–11 in coronal, sagittal, and axial images.

The results of MEG were shown in Table 1 and Figures 1–3. Clusters of SECD were displayed in inferior frontal regions in five patients (Patient 1, 3, 4, 5, and 6), temporal-insular regions in two patients (Patient 7 and 9), frontal-temporal-insular regions in two patients (Patient 8 and 11), parietal-insular regions in one patient (Patient 10), and sparsely dipoles were shown in central regions in one patient (Patient 2).

Based on the semiology and the results of the non-invasive evaluation, all patients had intracranial electrodes (subdural electrodes or stereo-EEG) implantation including insula/insular opercular regions (Supplementary Materials showed invasive EEG results of Patient 3, 5, and 6). The results showed seizure start in insular region in three patients (Patient 3–5), insular and hippocampal regions in two patients (Patient 1 and 2), frontal/temporal/parietal opercular regions including insula in four patients (Patient 6, 7, 8, and 11), and frontal/temporal opercular regions in two patients (Patients 9 and 10).

### MAP Findings

**Figure 4:**
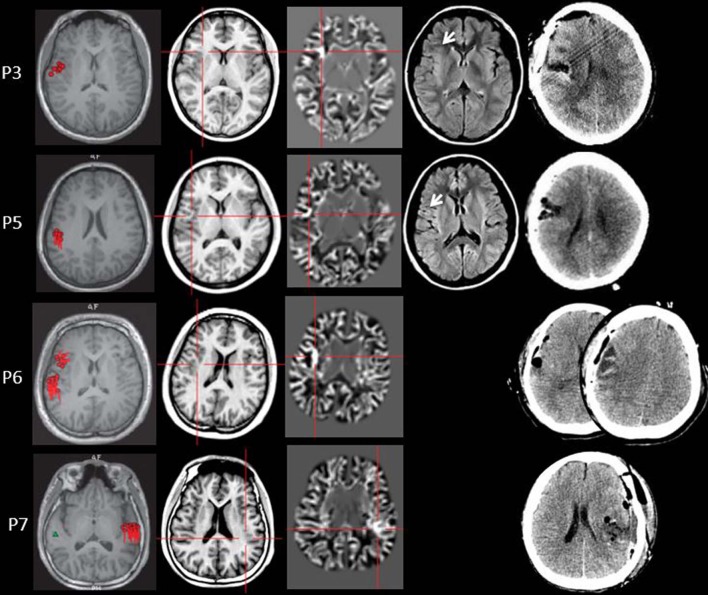
The MEG, MAP, and post-surgical results of patients with positive MAP results (Patient 3, 5, 6, and 7). The crosshairs pinpoint the location of the MAP-positive region. First column: MEG results of Patient 3, 5, 6, and 7. Second column: T1-weighted magnetization prepared rapid acquisition with gradient echo (MPRAGE) images used as input to MAP. Third column: gray–white matter junction z-score file as the output of MAP. Fourth column: T2-weighted fluid-attenuated inversion recovery (FLAIR) images of P3 and P5; the FLAIR images of P6 and P7 were not available. Fifth column: post-operative CT scan indicating site and extent of resection. Three patients with positive MAP results (Patient 3, 5, and 7) had complete resection of the MAP-positive regions, and remained seizure-free at 12 months after surgery. One patient (Patient 6) had incomplete resection of the MAP-positive regions and this patient still had seizure at 12-month follow-up. Pathology: P3, FCD IIb; P5, FCD IIa; P6, FCD IIa; P7, FCD IIb.

**Figure 5:**
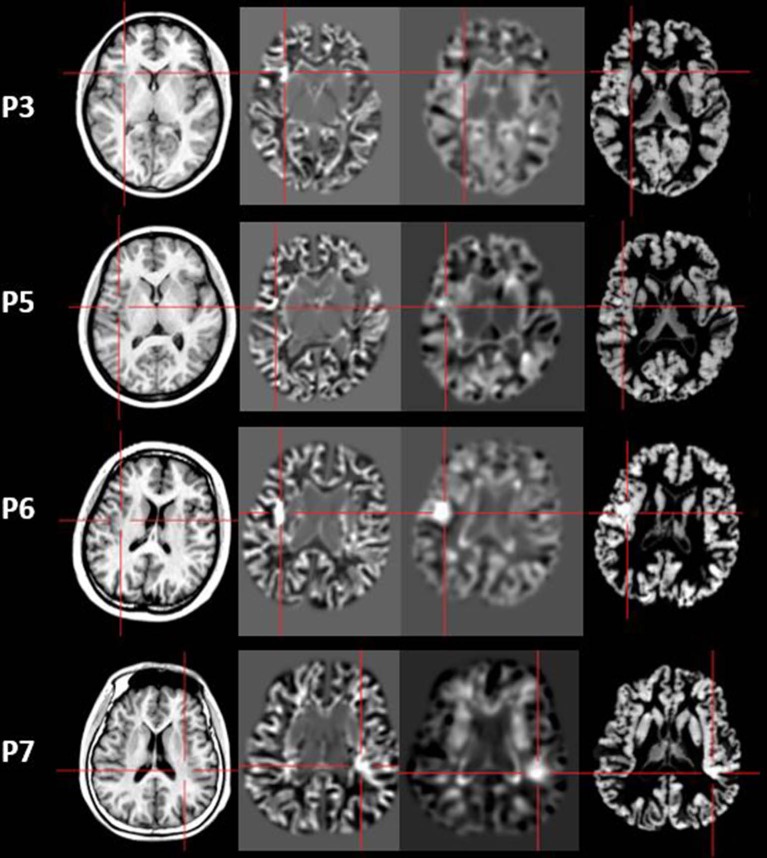
The junction z-score images, extension z-score images, and thickness z-score images of MAP-positive patients (Patient 3, 5, 6, and 7). First column: T1-weighted magnetization prepared rapid acquisition with gradient echo (MPRAGE) images used as input to MAP. Second column: junction z-score image of MAP. Third column: extension z-score image of MAP. Fourth column: thickness z-score image of MAP. All of the four patients had positive results on junction z-score images. However, only two patients (P6 and P7) showed positive results on extension z-score images and thickness z-score images.

Four out of 11 patients had positive MAP results (Patients 3, 5, 6, and 7). All of the four patients showed positive results on junction image, and two patients showed positive results on extension image and thickness image (Patient 6 and 7). The other patients showed negative results on junction image, extension image and thickness image. The results of the positive MAP findings were shown in Figures 4, 5. And we also provided one example of different z-score threshold (z-score = 1, 2, 3, and 4) we used to identify MAP positive regions (Patient 7, Supplementary Material). Overall, the positive rate was 36.4%. The positive regions were in insular (Patient 3) or operculoinsular regions (Patient 5, 6, and 7).

### Surgical Outcomes and Pathology

All of the 11 patients received surgeries including operculoinsular regions. Patient 8 had a second resection surgery after the first surgery failed. The mean follow-up period is 51.5 months of this cohort and all patients had a follow-up over 12 months. Nine out of 11 patients (82%) were seizure-free at the end of the last interview (Engel's class Ia). The surgical pathology showed FCD I in four patients, FCD IIa in five patients and FCD IIb in two patients. Three of the four patients became seizure-free following resection of MAP-positive and MEG-positive regions (histopathology showed FCD IIb in two patients and FCD IIa in one patient); one patient had worthwhile seizure reduction after surgery (Patient 6). Three patients (Patient 1, 2 and 8) had resection surgeries including hippocampus and none of them showed hippocampal sclerosis. Four patients had resection surgeries including temporal neocortex, and FCD I presented in two patients (Patient 1 and 11). Patient 2 showed FCD IIa and Patient 8 only showed mild microglial cell proliferation in temporal neocortex.

## Discussion

Because of the wealth of insular connections to surrounding lobes, the clinical manifestations of operculoinsular seizures are diverse (7, 28). Therefore, the localization of the seizure onset zone in some cases can be quite difficult and this is also a major reason of some surgery failures (29–31). On one side, the insula can generate viscerosensory symptoms and gastromotor symptoms such as abdominal sensation that may mimic temporal lobe seizures (8, 9). On the other side, it can produce somatosensory symptoms such as paresthesias aura of the limbs or the midline (13, 32), and it also have been reported to have complex motor behavior that is reminiscent of frontal lobe seizures (10). While the clinical features of operculoinsular epilepsy are diverse, some red flags have been identified to suspect insula involvement, such as perisylvian seizures, temporal plus epilepsy, hypermotor seizures, MRI-negative frontal and parietal lobe epilepsies, and insular lesions (33).

In patients with operculoinsular epilepsy, the information from the non-invasive examination is very limited, especially when MRI is negative (33). In our patients, scalp interictal EEG can be normal or showing interictal discharges in frontal, temporal, central or parietal region. Similarly, ictal EEG could show an onset arising from frontal central or temporal regions. MEG has also been reported as a tool in presurgical workup of temporo-perisylvian-insular epilepsy. It is especially useful if a tight dipole cluster is identified, even if other non-invasive workups are unremarkable (34–36). The preoperative MRI of all patients in the present study was negative. However, the results of MEG showed abnormalities in inferior frontal, temporal or central regions in all the patients. Alomar et al. reported that in 39 patients without insular semiology, 15 patients had MEG abnormalities located in the insula, while only 8 patients showed MRI abnormalities of the insula (6).

Because of the above-mentioned reasons, stereo-EEG is often mandatory to determine the precise seizure onset zones of insular epilepsy, in particular when MRI proves to be negative (13, 28, 33). The frequently used techniques can be orthogonal, oblique, or a combination of both trajectories (6). Of course, the implantation of the intracranial electrodes can have some other benefits, such as spare part of the mesial temporal and decrease the risk of the post-operative memory alteration when the symptoms of temporal plus epilepsy was presented (37). The ictal patterns on stereo-EEG usually consist of low-voltage fast recruiting activity, evolving into rhythmic high-frequency spikes, often limited to one quadrant of the insula (5, 13, 38).

Nevertheless, considering the cost and risk of implanting electrodes within the deep-seated and highly vascularized insula, it would be preferable to identify the subset of patients for whom insular intracerebral electrodes would best yield results. As MAP is non-invasive, low-cost and easy-handled, it can be used as a supplementary tool to other presurgical evaluation methods in the process of the detection of the seizure onset zone (39, 40). Previous reports have shown MAP can help detect the epileptogenic zone in the frontal, temporal, parietal, occipital regions (18, 22, 41, 42), with a sensitivity of 0.9 and specificity of 0.67 (18). The lesion can be either unifocal or multifocal (18). However, the report of the applications of MRI post-processing methods used in insular or operculoinsular epilepsy is relatively rare. As operculoinsular epilepsy is commonly MRI negative (28), the application of non-invasive examinations which can help improve the detection rate of the epileptogenic lesion appears to be more important.

Overall, the MAP-positive rate was 36.4% in our patients. This is a little lower than previous studies containing a mixed cohort of all types of seizures (18). The seizure-free rate (75%) of our cohort is higher than the previously reported insular epilepsy surgeries, which reported to be around 33.3–62.5% (6, 43, 44). These may due to the small sample size and the large resection range in the present study. As for the pathology of the MAP-positive patients, two patients were FCD IIa and two patients were FCD IIb. Previous studies showed that the pathology results of MAP positive regions can be FCD I, FCD II, as well as FCD III (18, 27). FCD II are highly epileptogenic lesions frequently causing pharmacoresistant epilepsy. Even in a specialized tertiary epilepsy center, 14% of all FCD II lesions remained undetected by conventional visual analysis, and by using morphometric analysis, the detection rate of FCD can be improved, especially in FCD IIa (22, 41). It is known that FCD I is less focal than other FCD subtypes (45, 46), therefore, we assumed that MAP may not be able to accurately delineate the FCD I lesions than FCD II lesions. One patient with positive MAP did not achieve seizure freedom after surgery. Patents 6 were thought to have functional areas involved at the presurgical evaluation conference, therefore, the resection strategy spared some functional areas in order to diminish the injure; and this patient also had some improvement after surgery.

Several limitations should be mentioned in the present study. Firstly, the major limitation of this study was the number of patients studied was relatively small. As operculoinsular epilepsy is often difficult to recognize and the MRI is often non-lesional, this pilot study suggested MAP may have a role identifying insular cases. Secondly, given the retrospective nature of this study, selection bias might exist. Patients with relatively focal epileptogenic lesions were more likely to receive surgery, and thus end up being selected in our study. Further studies using larger samples are required to confirm our hypothesis and to explore the non-invasive post-processing methods in the detection of the lesions in the operculoinsular regions. Besides, MAP analysis may have false positive results sometimes. According to previous studies, false positive rate is around 2% in normal controls (18). In epilepsy patients, the false positive rate may be higher because there are different kinds of artifacts that may be difficult to be distinguished from real lesions. We summarized common artifacts in MAP analysis in another paper (27), including motion artifacts, banding artifacts, bias field artifacts, vessels, white matter inhomogeneity, enlarged perivascular space and pulsation artifact from artery close by. MEG findings do help to exclude false positives sometimes (20, 47). However, we didn't use MEG results to exclude false positives in this study. In order to decrease the false positives, all the MAP results were reviewed by two independent reviewers in the present study.

## Conclusions

MAP is a useful tool in detection the epileptogenic lesions in patients with operculoinsular epilepsy. The overall MAP-positive rate was 36.4% in the present study. FCD type II may be more detectable than FCD type I in operculoinsular epilepsy cases. Notably, in order to make a right surgical regime decision, MAP results should always be interpreted in the context of the patient's anatomo-electroclinical presentation and large samples size studies are required to further explore the application of MRI post-processing methods in operculoinsular epilepsy patients.

## Data Availability Statement

All datasets generated for this study are included in the article/Supplementary Material.

## Ethics Statement

The studies involving human participants were reviewed and approved by Research Ethics Committee of Xuanwu Hospital, Capital Medical University. Written informed consent to participate in this study was provided by the participants' legal guardian/next of kin.

## Author Contributions

WW: study design, data analysis, VBM analysis, and manuscript writing. QZ: clinical data collecting. XZ: MEG data analysis. LL: scalp EEG data analysis. CX: SEEG information collecting. YP: pathology data review. SW: MEG data analysis. YaW: pathology data review. ZZ: MRI data collecting. WD: SEEG information collecting. YL: study design and manuscript review. YuW: study design.

### Conflict of Interest

The authors declare that the research was conducted in the absence of any commercial or financial relationships that could be construed as a potential conflict of interest.
