# Supplementary material for: Pilot Study of Voxel-Based Morphometric MRI Post-processing in Patients With Non-lesional Operculoinsular Epilepsy
Source: Front Neurol. 2020 Mar 19;11:177. doi: 10.3389/fneur.2020.00177 (PMC7096577; doi:10.3389/fneur.2020.00177)
Supplement: Supplementary file 1 [file Data_Sheet_1.pdf]

Supplementary Figure 1. Invasive EEG results of Patient 3

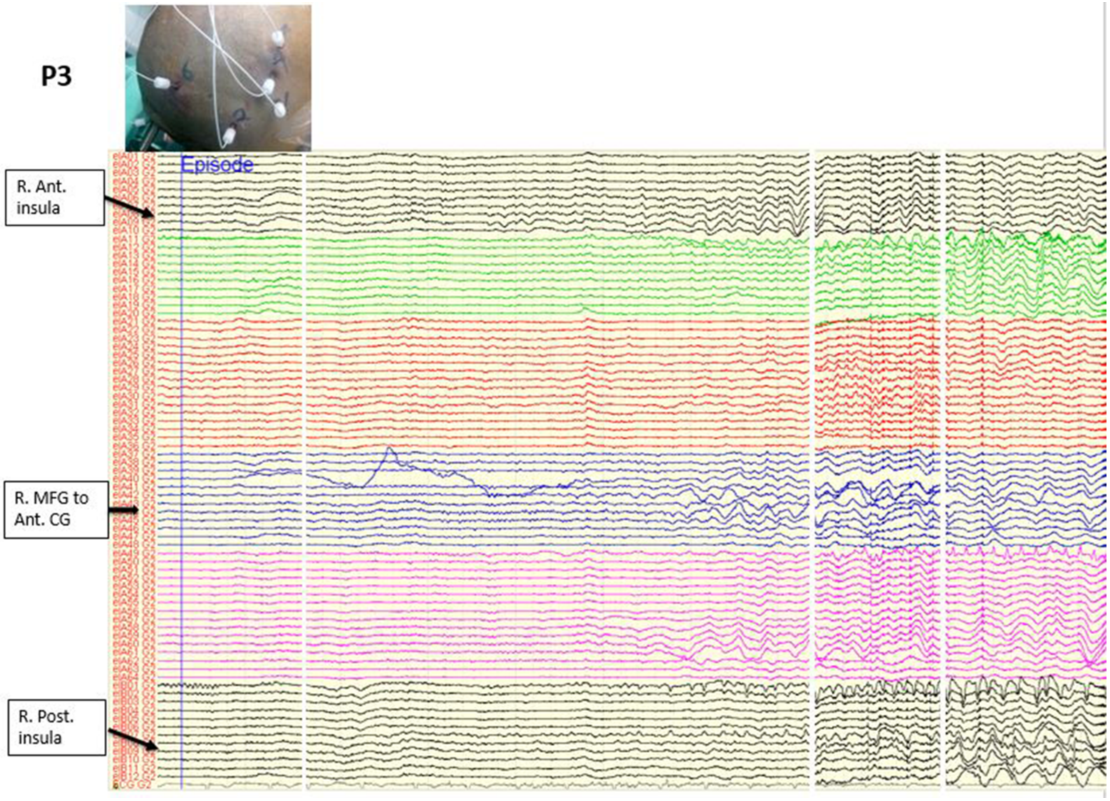

Supplementary Figure 2. Invasive EEG results of Patient 5

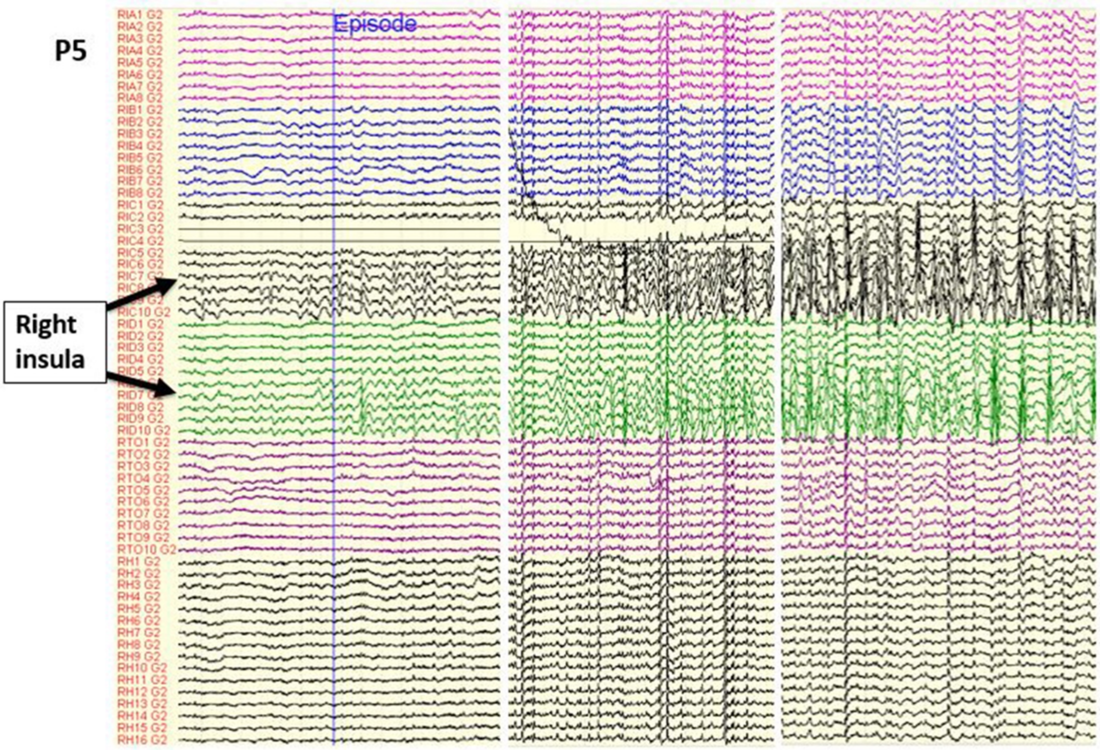

Supplementary Figure 3. Invasive EEG results of Patient 6

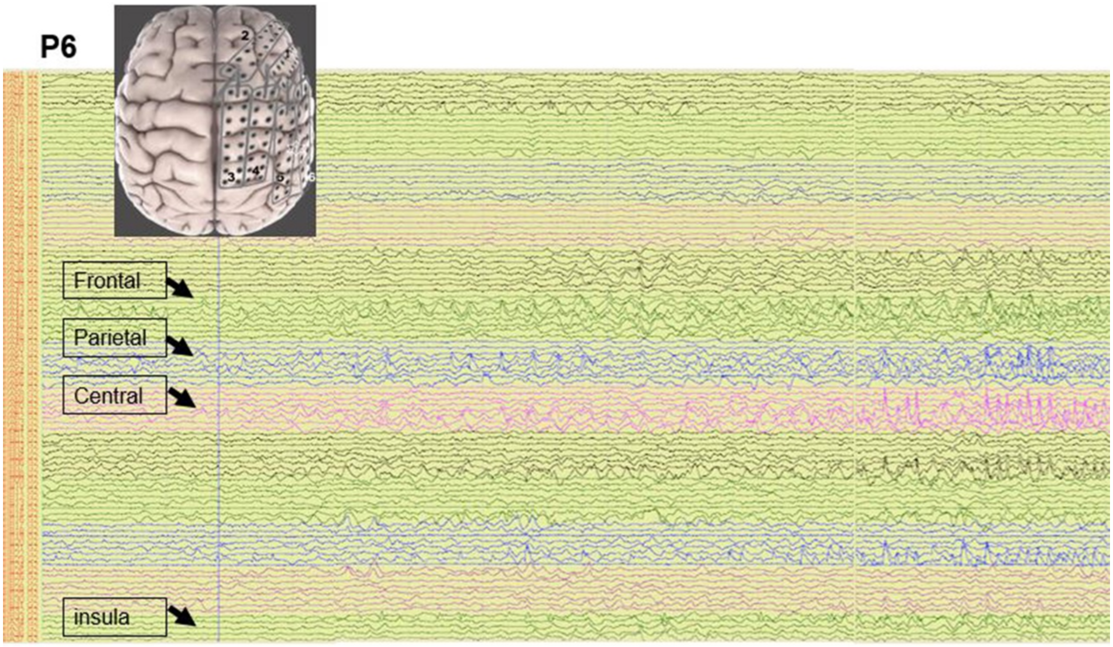

Supplementary Figure 4. Different z-score threshold (z-score = 1, 2, 3 and 4) we used to identify MAP positive regions (Patient 7)

Z-score	T1	Junction image	Thickness image	Extension image
z-score = 1<br>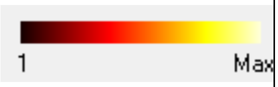	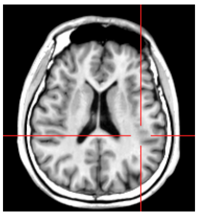	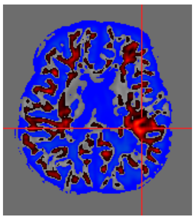	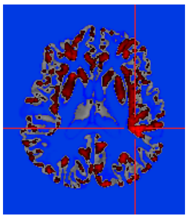	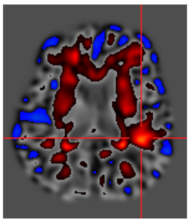
z-score = 2<br>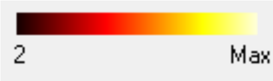	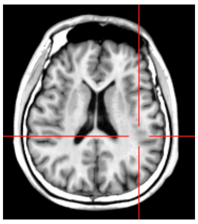	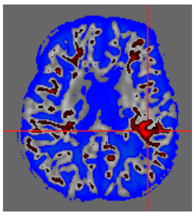	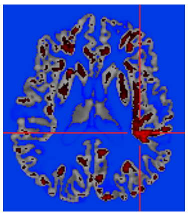	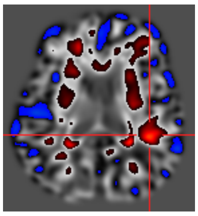
z-score = 3<br>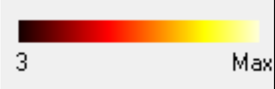	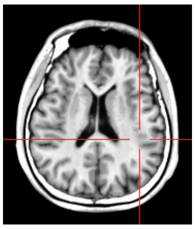	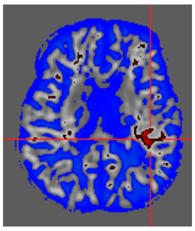	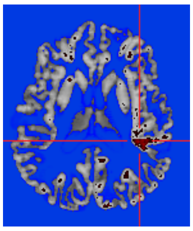	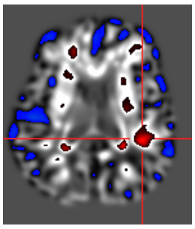
z-score = 4<br>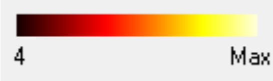	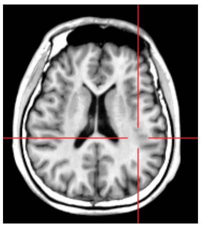	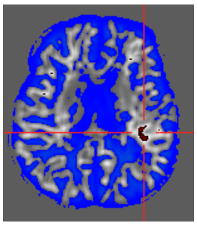	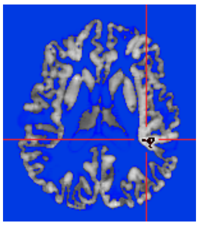	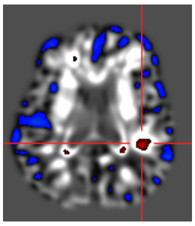
